# Molecular basis for the activation of the bitter taste receptor TAS2R14 by Ritonavir

**DOI:** 10.1371/journal.pone.0332704

**Published:** 2025-09-19

**Authors:** Jiao Wen, Xinyi Ma, Xinyi Zhou, R. Charles Kissell, Yongcheng Lu, Yukyoung Kim, Young Seo Lee, Alice Lee, Shurui Chen, Keman Xu, Leigh D. Plant, Meng Cui

**Affiliations:** Department of Pharmaceutical Sciences and the Center for Drug Discovery, School of Pharmacy and Pharmaceutical Sciences, Bouvé College of Health Sciences, Northeastern University, Boston, Massachusetts, United states of America; University of Coimbra: Universidade de Coimbra, PORTUGAL

## Abstract

Ritonavir is a protease inhibitor used in combination with other antiretroviral drugs to treat HIV, especially in children. It enhances the effectiveness of these drugs by inhibiting the cytochrome P450-3A4 enzyme, thereby increasing their bioavailability. Ritonavir is also being investigated for cancer treatment due to its mechanism of action. However, its intense bitterness, particularly in liquid formulations, can be intolerable for some children. This bitterness is attributed to its activation of bitter taste receptors, including TAS2R14 (also named T2R14), as demonstrated in our previous study. In this study, we utilized molecular modeling, site-directed mutagenesis, and cell-based calcium mobilization assays to characterize the key residues involved in TAS2R14 activation by ritonavir. Eight critical residues for ritonavir interacting with the receptor were discovered. The results indicate two potential binding sites for ritonavir in TAS2R14 receptor, including orthosteric and allosteric sites. These findings can be useful for developing bitter blockers targeting TAS2R14 to eliminate or reduce the bitter taste of ritonavir.

## Introduction

Ritonavir is an antiretroviral medication used in the treatment of HIV (Human Immunodeficiency Virus), particularly in children. It belongs to protease inhibitors. Ritonavir works by inhibiting the HIV protease enzyme required for viral replication [1]. It is often used in combination with other antiretroviral drugs as part of highly active antiretroviral therapy (HAART) to boost their pharmacokinetic profiles and enhance overall effectiveness [2]. Ritonavir is also being investigated for cancer treatment due to its mechanism of action [3]. However, ritonavir is intensely bitter and is typically available in liquid formulations, which can be unbearable for some children [4]. According to previous studies, the bitter sensory recognition threshold for ritonavir is only 0.0702 mM [5], yet the concentration used in the HIV drug KALETRA is 28 mM. Ritonavir activates multiple human TAS2Rs, including TAS2R1, TAS2R8, TAS2R13, and TAS2R14, with EC_50_ values in the micromolar range, as demonstrated by our previous research [6].

TAS2R14, encoded by the TAS2R14 gene which is located on chromosome 12p13 in the taste receptor gene cluster in humans, is a bitter taste receptor that belongs to the family of G-protein-coupled receptors [7]. TAS2R14, like other bitter taste receptors, works by binding to bitter compounds and activating intracellular signaling pathways that lead to the perception of bitterness. This signaling typically results in the release of intracellular calcium [8]. These receptors are primarily expressed in the taste receptor cells of the tongue and palate epithelia and are crucial for identifying potentially harmful substances, as many toxic compounds have a bitter taste. But they are also found in other tissues, including the respiratory system and immune cells [9]. TAS2R14 plays a role in modulating innate immune responses. It has been shown to be expressed in various immune cells, such as monocytes and macrophages, and its activation can lead to increased nitric oxide production and enhanced phagocytosis of bacteria like *Escherichia coli* and *Staphylococcus aureus* [10]. In the respiratory system, TAS2R14 is expressed in the smooth muscle of human airways, where its activation induces bronchodilation by increasing intracellular calcium ion concentration and activating potassium channels [11]. Additionally, TAS2R14 has been identified as a potential therapeutic target for upper respiratory infections, with flavones shown to activate it and modulate respiratory epithelial innate immunity [12]. T2R14 has also been identified in breast and pancreatic cancer cells, where it exhibits antiproliferative and anti-migratory effects. In pancreatic cancer, high expression of T2R14 has been associated with longer patient survival, suggesting a potential functional role in cancer progression and treatment [13]. Overall, T2R14 is a versatile receptor with significant roles in taste perception, immune response, respiratory health, and potentially cancer therapy.

The structure of the human bitter taste receptor TAS2R14 has been elucidated using cryo-electron microscopy (cryo-EM), revealing key details about its conformation and ligand interactions [14–17]. T2R14 is highly promiscuous, responding to a wide variety of chemically diverse agonists. One of cryo-EM structures of TAS2R14 shows it in complex with its signaling partner gustducin and bound to Flufenamic acid, a clinically approved drug [16]. TAS2R14, like other members of the TAS2R family, consists of seven transmembrane alpha-helices that span the cell membrane. These helices form a bundle that is crucial for the receptor’s ability to interact with bitter compounds and transduce signals inside the cell. The structure reveals significant changes in the orientation of TMs 2, 4, 5, and 7 when compared to other related receptors like TAS2R46. Notably, there is an outward movement of TM6 in TAS2R14 [16]. The receptor has multiple ligand-binding sites, including an intracellular binding pocket (allosteric site). This pocket accommodates various ligands, indicating a unique activation mechanism. The structure also shows a cholesterol molecule occupying the orthosteric site, which is common in class A GPCRs [14,15,17]. TAS2R14 interacts with different G protein subtypes, including gustducin and Gαi2 proteins. These interactions are crucial for its signaling and activation pathways. The receptor’s ability to bind multiple ligands and interact with various G proteins highlights its broad-spectrum recognition capabilities. These studies reveal the arrangement of the transmembrane helices and the binding sites for ligands, enhance our understanding of bitter taste perception and provide a foundation for developing therapeutic applications targeting TAS2R14.

In this study, we tested 39 mutations in both the orthosteric and allosteric sites of the TAS2R14 receptor for their responses to ritonavir using a calcium mobilization functional assay, identified key residues involved in ritonavir binding. Our results can be useful for developing bitter blockers targeting TAS2R14 to eliminate the bitter taste of ritonavir.

## Materials and methods

### Molecular modeling and simulations

The hTAS2R14 receptor cryo-EM structure (PDBID:8XQO) was prepared by the Protein Preparation Wizard module of Maestro (2024−3) program (Schrödinger, Inc.). The cholesterol in the orthosteric site and Aristolochic acid in the allosteric site of TAS2R14 were used as reference, induced-fit-docking (IFD) simulations [18] were conducted. The residues within 5Å of ligand poses were selected for side chain optimization by prime refinement. The XP scores were used for ranking of the ligand poses, and the top 20 poses of docked ligand were saved for visual inspection and selection. The poses of the docked ligands with the best XP docking scores were selected as the predicted binding conformation. Ligand and receptor interactions were analyzed by using Discovery Studio 2017 (Dassault Systèmes).

### Materials

Aristolochic acid and Flufenamic acid were purchased from Sigma. Oleuropein aglycon, was isolated in the Magiatis lab from olives or olive oil as previously described [19]. Polyethylenimine (PEI) was obtained from Polysciences, Inc.. Poly-D-lysine (PDL) was purchased from Alamanda Polymers Inc. Ritonavir was purchased from Cayman Chemical. Penicillin streptomycin, Gibco HBSS buffer, and Gibco 1M HEPES (4-(2-hydroxyethyl)-1-piperazineethanesulfonic acid) buffer were obtained from Life Technologies. Fetal bovine serum, and Opti-MEM reduced serum medium were also obtained from Life Technologies. DMEM high glucose medium was purchased from Thermo Fisher Scientific. 96-well plates for cell culture were purchased from Greiner Bio-One.

### Molecular biology and cell culture

Amino Acid substitutions were introduced into the T2R14 gene carried by pcDNA 3.1 vector as described previously [19]. Site-directed mutagenesis of T2R14 was done by PCR-mediated recombination. DNA sequences of all the mutations were verified by DNA sequencing (Psomagen). The wild type T2R14 and mutant receptor genes were transiently transfected in HEK293E cells using PEI according to previously published methods [6]. Approximately 75000/well HEK293E cells were seeded onto 96-well PDL-coated plates and cultured at 37°C overnight, the medium was changed to Opti-MEM supplemented with 5% FBS (filtered) before transfection, then receptor DNA (0.08 μg/well) was transfected together with Gα16-gust44 (0.08 μg/well) and the GCaMP calcium sensor (0.05 μg/well) using PEI (1:8 for total DNA and PEI). Following 4 hours after transfection, the medium was replaced with OPTI-MEM + 5% FBS (filtered) and contained 1% penicillin streptomycin.

### Calcium mobilization functional assay

The measurement of intracellular calcium produced was carried out as follows. Following an additional 44 hours, the medium was replaced with 50 μl Hank’s Buffered Salt Solution (HBSS) supplemented with 20 mM HEPES (HBSSH) and incubated around 20 min. Receptor activation was determined by measuring changes in intracellular calcium by monitor fluorescence changes after application of 50 μl tested compounds (tested compounds were dissolved in DMSO at 2x concentration and diluted by HBSSH) using Flexstation3 fluorescence plate reader (Molecular Devices) at 525 nm emission following excitation at 488 nm, cutoff at 515 nm. The compounds were added at 30 s and readings were continued to an additional 150 s recorded every 2 s. Calcium mobilization in response to test compounds was quantified as the percentage change in fluorescence, calculated as (ΔF/F), where ΔF is the difference between peak and baseline fluorescence levels, and F is the baseline fluorescence. Data were collected as triplicates and 6 times as biological replicates. All the results were tested under 25ºC. Statistics analysis was performed by using one-way ANOVA Tukey’s multiple comparison test in Prism 10 (GraphPad Software Inc., San Diego, CA). Data represent mean ± SEM.

### Surface expression

Total internal reflection fluorescence (TIRF) microscopy was used to selectively illuminate membrane-proximal fluorescence [20]. Following transient expression of GFP-tagged TAS2R14 or mutant constructs in HEK293T cells, images of fluorescent cells were analyzed in ImageJ. Background subtraction was performed using a rolling ball radius of 50.0 pixels. Regions of interest (ROIs) were identified by a ‘Triangle’ thresholding procedure, in which a line is drawn on a histogram of intensity values from peak to minimum frequency, and the threshold is defined as the intensity value on the histogram at the longest distance from this line [21]. Contiguous regions larger than 250 square pixels that exceeded this threshold were identified as ROIs. The average intensities of these regions were compared between all GFP-tagged mutants and GFP-tagged wild-type T2R14 to assess relative membrane expression efficiency.

## Results

### Computational prediction of ritonavir binding sites in TAS2R14

**Fig 1:**
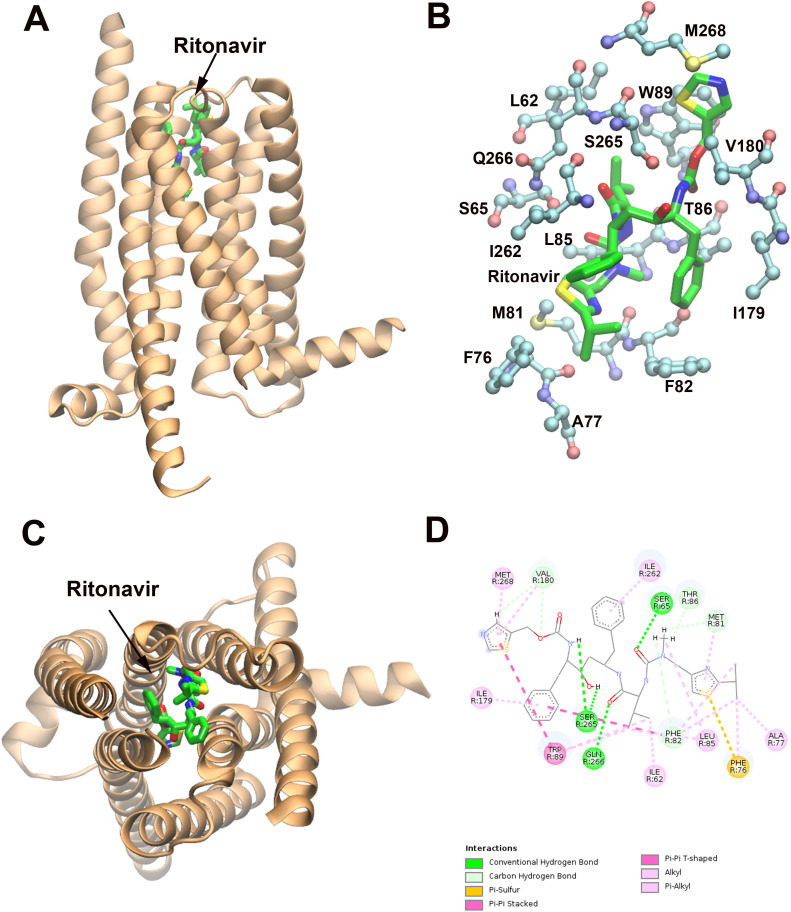
Ritonavir-hTAS2R14 complex model (upper (orthosteric) site) predicted by Induced Fit Docking (IFD). **A.** The predicted binding site of ritonavir in hTAS2R14 (side view). hTAS2R14 (PDBID:8XQO) is rendered as newcartoon (in orange). Ritonavir is depicted in licorice colored by atom types. **B.** Predicted ritonavir upper binding site in hTAS2R14. The binding site residues and ritonavir are represented in ball-stick and licorice, respectively, and colored by atom types. **C.** top (from extracellular side) view of **A. D.** 2D plot (by Discovery Studio) of the detailed interactions between ritonavir and hTAS2R14 receptor binding site residues.

To explore the potential binding sites of ritonavir in TAS2R14, we conducted induced fit docking (IDF) simulations using Schrödinger software (see Methods). We selected a cryo-EM structure of TAS2R14 (PDBID:8XQO), which was solved in complex with cholesterol (orthosteric site), and Aristolochic acid (allosteric site), for the IFD simulations. We used cholesterol and Aristolochic acid as reference sites for IFD, and docked ritonavir into orthosteric (upper) and allosteric (lower) sites, respectively. **Fig 1** shows ritonavir docked into the orthosteric (upper) site of TAS2R14. We used Discovery Studio 2017 for the ligand and receptor interaction analysis. Key ritonavir interacting residues include S65^2.60^, T184^5.44^, S265^7.38^, and Q266^7.39^, which form hydrogen bonds; F82^3.25^ and W89^3.32^, which engage in pi-pi interactions; and L62^2.57^, F76, A77, M81^3.24^, L85^3.28^, T86^3.29^, I179^5.39^, V180^5.40^, I262^7.35^, and M268^7.41^, which participate in hydrophobic interactions (Fig 1B and 1D) (generic residue numbers of hTAS2R14 were obtained from https://gpcrdb.org/family/009_001_001_010/) [22].

**Fig 2:**
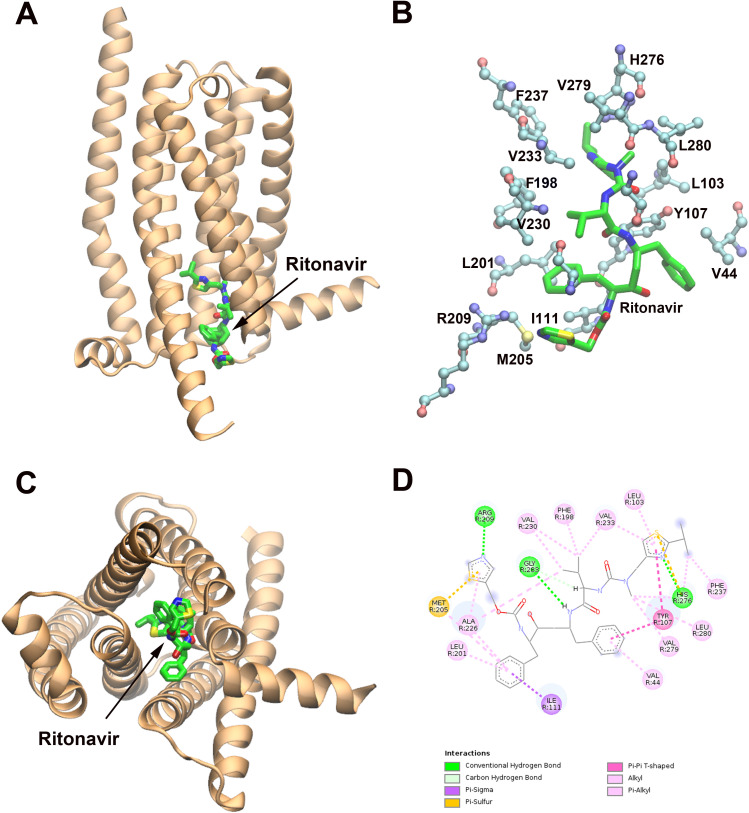
Ritonavir-hTAS2R14 complex model (lower (allosteric) site) predicted by Induced Fit Docking (IFD). **A.** The predicted binding site of ritonavir in hTAS2R14 (side view). hTAS2R14 (PDBID:8XQO) is rendered as newcartoon (in orange). Ritonavir is depicted in licorice colored by atom types. **B.** Predicted ritonavir upper binding site in hTAS2R14. The binding site residues and ritonavir are represented in ball-stick and licorice, respectively, and colored by atom types. **C.** top (from extracellular side) view of **A. D.** 2D plot (by Discovery Studio) of the detailed interactions between ritonavir and hTAS2R14 receptor binding site residues.

Fig 2 shows ritonavir docked into the allosteric (lower) site of TAS2R14. Key ritonavir interacting residues include R209^5.69^, H276^7.49^, and G283^7.56^, which form hydrogen bonds; Y107^3.50^, which engage in pi-pi interactions; V44^2.39^, L103^3.46^, I111^3.54^, L201^5.61^, M205^5.65^, V233^6.38^, F237^6.42^, V279^7.52^, L280^7.53^, F298^8.61^, which participate in hydrophobic interactions (Fig 2B and 2D).

### Mutagenesis studies of predicted binding site residues using an Alanine scan

We used mutagenesis and calcium mobilization assay to test wild type TAS2R14 and its mutations’ function. Guided by a molecular model of the receptor and its interaction with ritonavir, we introduced mutations by substituting key residues with the smaller amino acid Alanine. Our hypothesis is that this substitution will minimally impact receptor folding and ligand binding. Functional analysis of wild type and mutant TAS2R14 receptors was determined by measuring changes in intracellular calcium of HEK293E cells transiently transfected by these receptors after application of bitter tasting drugs, 30 μM Aristolochic acid (AA), 100 μM ritonavir, 30 μM Flufenamic acid (FA) and 100 μM oleuropein aglycon (OA).

### Mutations in the orthosteric site of TAS2R14

**Fig 3:**
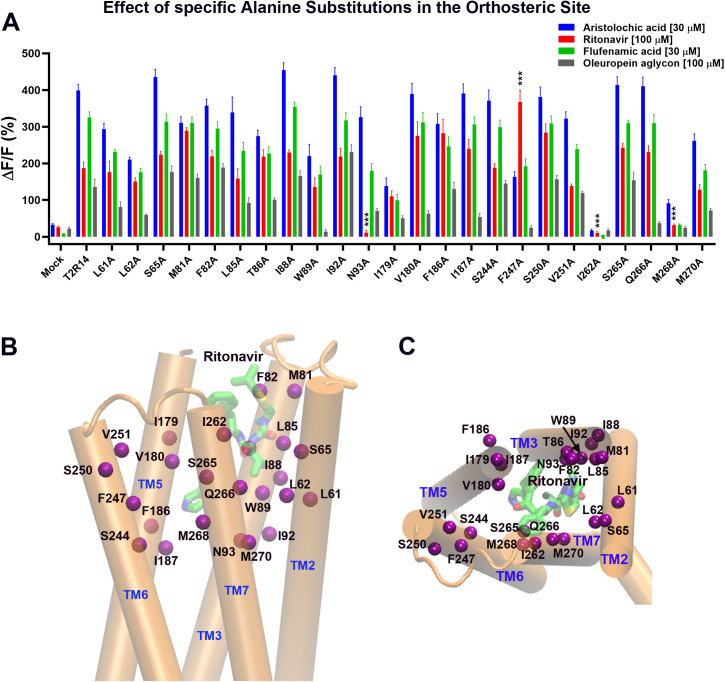
Point mutations of selected residues in the hTAS2R14 binding pocket (upper (orthosteric) site) affect response to ritonavir. **A.** Intracellular calcium signals induced by ritonavir (100 μM) and control compounds (30 μM Aristolochic acid (AA), 30 μM Flufenamic acid (FA) and 100 μM oleuropein aglycone (OA)) in HEK293E cells transfected with hTAS2R14 or mutants. The data presented were from at least six independent measurements (mean ± SEM). ΔF/F (%), percentage of relative fluorescence increase. Mock, without hTAS2R14 receptor transfected cells as a negative control. Asterisks indicate significant differences tested by one-way ANOVA (***p < 0.001, **p < 0.01, *p < 0.05, N ≥ 6). The figure was plotted by GraphPad. **B.** location of the upper binding site residues tested (Cα atom only in purple) in the hTAS2R14 (side view, cartoon in orange). The TM1 and TM4 were removed for clarity. **C.** the top view of **B**.

For the orthosteric (upper) site of TAS2R14, we mutated residues, L61^2.56^, L61^2.56^, S65^2.60^, M81^3.24^, F82^3.25^, L85^3.28^, T86^3.29^, I88^3.31^, W89^3.32^, I92^3.35^, N93^3.36^, I179^5.39^, V180^5.40^, F186^5.46^, I187^5.47^, S244^6.49^, F247^6.52^, S250^6.55^, V251^6.56^, I262^7.35^, S265^7.38^, Q266^7.39^, M268^7.41^, M270^7.43^ to Alanine, and tested their responses to the drugs (Fig 3). Fig 3B and 3C show the locations of these residues in the TAS2R14 structure model (TM2: L61^2.56^, L61^2.56^, S65^2.60^; TM3: M81^3.24^, F82^3.25^, L85^3.28^, T86^3.29^, I88^3.31^, W89^3.32^, I92^3.35^, N93^3.36^; TM5: I179^5.39^, V180^5.40^, F186^5.46^; TM6: S244^6.49^, F247^6.52^, S250^6.55^, V251^6.56^; TM7: I262^7.35^, S265^7.38^, Q266^7.39^, M270^7.43^). Fig 3A shows the percentage change in fluorescence upon drug addition to HEK293E cells transfected with either the wild-type TAS2R14 receptor or receptor mutants, together with the Gα16gust44 and GCaMP6s. As shown in the Fig 3A, cells without receptor expression (Mock) did not exhibit significant fluorescence changes in response to any of the tested drugs. In contrast, cells transfected with the TAS2R14 wild type responded well to all four drugs, with fluorescence signals increasing by approximately 400%, 190%, 330%, and 140%, for Aristolochic acid (30 μM), ritonavir (100 μM), Flufenamic acid (30 μM) and oleuropein aglycon (100 μM), respectively. The mutant I262A^7.35^ did not show responses to any of the tested drugs, indicating it may have lost its function. TIRF experiments show that the expression level of the I262A^7.35^ mutant is comparable to that of the wild-type receptor (S1 Fig). Mutants N93A^3.36^ and M268A^7.41^ did not respond to ritonavir, while N93A^3.36^ responded to the other tested drugs, and M268A^7.41^ responded only to Aristolochic acid with weaker signal compared to the wild-type receptor. Interestingly, F247A^6.52^ showed an increased response to ritonavir, while decreased responses to other tested drugs. None of the mutants exhibited clearly reduced responses to either AA or FA, while they maintained strong responses to ritonavir or oleuropein aglycon. In contrast, mutants, W89A^3.^^32^, V180A^5.^^40^, I187A^5.47^, F247A^6.52^, Q266A^7.39^ showed either abolished or significantly reduced responses to oleuropein aglycon compared to their responses to other tested drugs.

### Mutations in the allosteric site of TAS2R14

**Fig 4:**
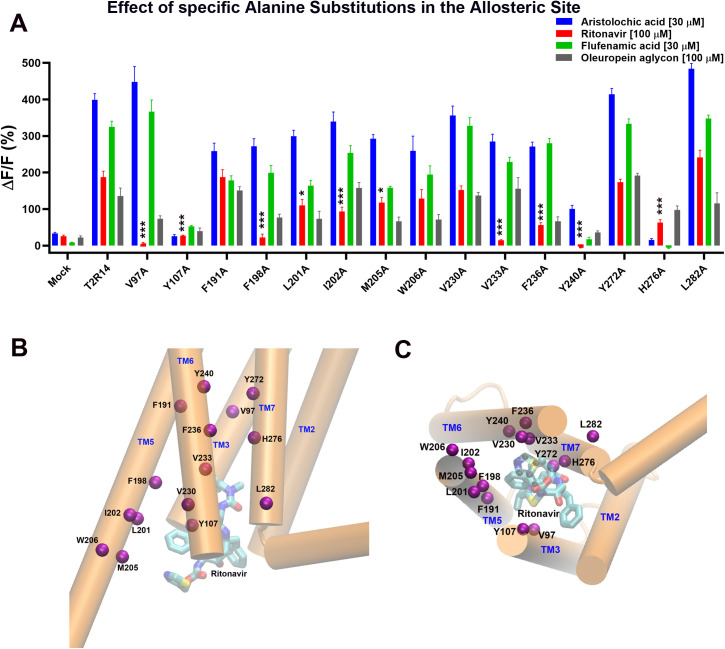
Point mutations of selected residues in the hTAS2R14 binding pocket (lower (allosteric) site) affect response to ritonavir. **A.** Intracellular calcium signals induced by ritonavir (100 μM) and control compounds (30 μM Aristolochic acid (AA), 30 μM Flufenamic acid (FA) and 100 μM oleuropein aglycone (OA)) in HEK293E cells transfected with hTAS2R14 or mutants. The data presented were from at least six independent measurements (mean ± SEM). ΔF/F (%), percentage of relative fluorescence increase. Mock, without hTAS2R14 receptor transfected cells as a negative control. Asterisks indicate significant differences tested by one-way ANOVA (***p < 0.001, **p < 0.01, *p < 0.05, N ≥ 6). The figure was plotted by GraphPad. **B.** location of the upper binding site residues tested (Cα atom only in purple) in the hTAS2R14 (side view, cartoon in orange). The TM1 and TM4 were removed for clarity. **C.** the top view of **B**.

For the allosteric site of TAS2R14, we mutated residues, V97^3.40^, Y107^3.50^, F191^5.51^, F198^5.58^, L201^5.61^, I202^5.62^, M205^5.65^, W206^5.66^, V230^6.35^, V233^6.38^, F236^6.41^, Y240^6.45^, Y272^7.45^, H276^7.49^ and L282^7.55^ to Alanine, and tested their responses to the four drugs (Fig 4). Fig 4B and 4C show the locations of these residues in the allosteric site of TAS2R14 (TM3: V97^3.40^, Y107^3.50^; TM5: F191^5.51^, F198^5.58^, L201^5.61^, I202^5.62^, W206^5.66^; TM6: V230^6.35^, V233^6.38^, F236^6.41^, Y240^6.45^; TM7: Y272^7.45^, H276^7.49^, L282^7.55^). From Fig 4A, we can see that mutants V97A^3.40^, F198A^5.58^, V233A^6.38^, F236A^6.41^, and Y240A^6.45^ exhibited abolished responses to ritonavir. Mutants L201A^5.61^, I202A^5.62^, and M205A^5.65^ showed slightly reduced responses to ritonavir compared to the wild-type receptor. Mutant H276A^7.49^ showed reduced responses to ritonavir and oleuropein aglycon, and abolished responses to Aristolochic acid and Flufenamic acid. Mutant Y240A^6.45^ showed reduced responses to Aristolochic acid and other control drugs compared with TAS2R14. Mutant Y107A^3.50^ did not respond to any tested drugs, which indicates it may lose function or a critical residue for all tested drugs to the receptor activation. TIRF experiments show that the expression levels of the Y107A^3.50^ and Y240A^6.45^ mutants are comparable to that of the wild-type receptor (S1 Fig). The rest mutants, F191A^5.51^, W206A^5.66^, V230A^6.35^, Y272A^7.45^, and L282A^7.55^ showed similar responses to all tested drugs compared to the wild-type receptor.

### Concentration-dependent effects on the key ritonavir interacting residues for ritonavir

**Fig 5:**
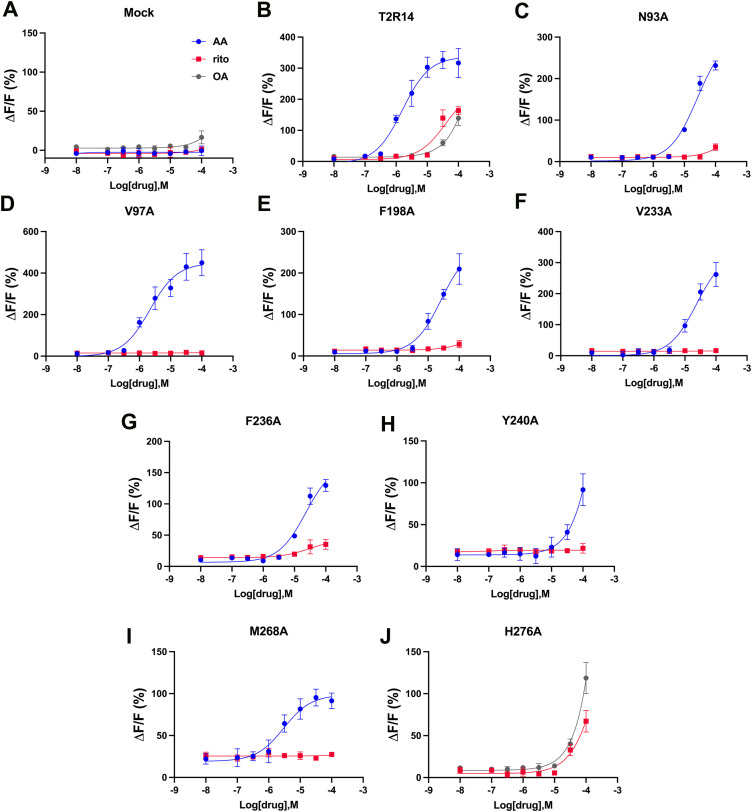
Dose-response relationships for hTAS2R14 mutants affect ritonavir activity. The Results were obtained using a cell-based assay of HEK293E cells co-transfected with hTAS2R14 or its mutants along with Gα16gust44 and GCaMP6s. Mock, without hTAS2R14 receptor transfected cells as a negative control. Each point represents the mean ± SEM from three independent measurements. The figures were plotted by Prism 10. ΔF/F (%), percentage of relative fluorescence increase.

To further validate the responses of these key mutants to the tested drugs, we conducted concentration-dependent activation assays of hTAS2R14 with these compounds. We tested eight different concentrations of each drug, ranging from 0.01 µM to 100 µM for AA, OA and ritonavir. Fig 5 depicts the concentration-dependent responses of the hTAS2R14 wild-type and mutants activated by ritonavir and AA/OA. Compared to the wild-type hTAS2R14, which responds to AA, OA and ritonavir in a dose-dependent manner, the mock control (without hTAS2R14 transfected) shows no response to any of the drugs. Mutants N93A^3.36^, N97A^3.40^, F198A^5.58^, V233A^6.38^, F236A^6.41^, Y240A^6.45^, and M268A^7.41^ respond to AA similarly (N93A^3.36^, V97A^3.40^, F198A^5.58^, and V233A^6.38^) or slightly reduced (F236A^6.41^, V240A^6.45^, and M268A^7.41^) to the wild-type receptor but show either an abolished (V97A^3.40^, F198A^5.58^, V233A^6.38^, Y240A^6.45^, and M268A^7.41^) or significantly reduced (N93A^3.36^ and F236A^6.41^) responses to ritonavir. Mutant H276A^7.49^ showed a reduced response to ritonavir compared to OA. The results indicate that these residues interact with ritonavir, and critical for ritonavir induced TAS2R14 activation. The results indicate that these residues interact with ritonavir and are critical for ritonavir-induced TAS2R14 activation.

## Discussion

Lopinavir/ritonavir (LPV/r) is the first-line treatment recommended by the World Health Organization for children aged three years and older who are infected with HIV. However, the bitterness of these drugs has been found to impact adherence in young children, affecting their survival [23]. Developing more child-friendly formulations of these protease inhibitors, particularly those with improved taste, is a critical need for children living with HIV. Our previous studies showed that anti-HIV drug ritonavir activates several hTAS2Rs, i.e., hTAS2R1, hTAS2R8, hTAS2R13 and hTAS2R14 [6]. Among them, hTAS2R14 is the most broadly tuned bitter taste receptor, which responds to a wide variety of chemically diverse bitter compounds [8]. Not only clinical drugs, bitter tasting phenolic compounds from olive oil, such as, ligstroside aglycon, and oleuropein aglycon also active TAS2R14 [19]. Therefore, understanding the molecular basis of the interaction between drugs like ritonavir and TAS2R14 would be helpful in developing bitter blockers to eliminate the bitter taste of these drugs. Given ritonavir’s ability to activate multiple bitter taste receptors, including T2R14 and others (T2R1/8/13), the use of a cocktail of receptor-specific blockers may be necessary to fully suppress its bitter taste or off-target signaling effects.

Recently, four different labs independently resolved the TAS2R14 structure using cryo-EM [14–17], providing an opportunity to predict how ritonavir interacts with the receptor using these high-accuracy experimentally determined structures. Interestingly, a cholesterol molecule was found occupying the orthosteric site of TAS2R14, while a bitter-tasting ligand binds to an allosteric site of the receptor [14,15,17]. Another TAS2R14 cryo-EM structure shows that the bitter-tasting drug Flufenamic acid can bind to both the orthosteric and allosteric sites in the absence of cholesterol [16]. An intriguing question is: where is the binding site for ritonavir in TAS2R14? Orthosteric, allosteric, or both?

Using IFD, we docked ritonavir into both orthosteric and allosteric sites in TAS2R14. Based on the model predictions, we selected some key interacting residues for Alanine scan mutagenesis studies. 39 residues in total (orthosteric site (24): L61^2.56^, L61^2.56^, S65^2.60^, M81^3.24^, F82^3.25^, L85^3.28^, T86^3.29^, I88^3.31^, W89^3.32^, I92^3.35^, N93^3.36^, I179^5.39^, V180^5.40^, F186^5.46^, I187^5.47^, S244^6.49^, F247^6.52^, S250^6.55^, V251^6.56^, I262^7.35^, S265^7.38^, Q266^7.39^, M268^7.41^ and M270^7.43^ (Fig 3B and 3C); Allosteric site (15): V97^3.40^, Y107^3.50^, F191^5.51^, F198^5.58^, L201^5.61^, I202^5.62^, M205^5.65^, W206^5.66^, V230^6.35^, V233^6.38^, F236^6.41^, Y240^6.45^, Y272^7.45^, H276^7.49^, and L282^7.55^ (Fig 4B and 4C) were selected to be mutated to Alanine, and tested using calcium mobilization assay. Eight mutants (orthosteric site: N93^3.36^ and M268^7.41^; allosteric site: V97^3.40^, F198^5.58^, V233^6.38^, F236^6.41^, Y240^6.45^, and H276^7.49^) showed clear effects on ritonavir but not on some control compounds, exhibiting either abolished or significantly reduced responses compared to the wild-type TAS2R14 receptor (Fig 3A and Fig 4A). The results were further validated through concentration-dependent experiments on these mutants (Fig 5).

In our study, we selected AA, FA, and OA, as control compounds to validate the function of the wild-type receptor and mutants, ensuring that the identified critical interacting residues were not due to loss-of-function mutations. We hypothesize that although ritonavir can bind to the same site on the receptor, it may interact with binding site residues differently compared to the control compounds. Indeed, not only did we identify the critical residues for ritonavir activation, but we also identified residues essential for the control compounds. For example, mutants W89A^3.32^, F247A^6.52^, and Q266A^7.39^ (orthosteric site) showed abolished responses to OA but exhibited normal responses to ritonavir compared to the wild-type receptor. Mutants F247A^6.52^ (orthosteric site) and H276A^7.49^ (allosteric site) showed abolished response to AA but exhibited reduced response to ritonavir. Additionally, mutants Y240A^6.45^and H276A^7.49^ (allosteric site) showed abolished responses to FA, while exhibiting reduced responses to AA or ritonavir (Fig 3 and 4. Previous studies showed that the mutants Y240A^6.45^, H276A^7.49^, H276F^7.49^, and H276R^7.49^ exhibited abolished responses to FA and AA [15,16], which are consistent with our results.

However, several of the T2R14 mutants examined in this study may influence receptor function through mechanisms beyond direct ligand binding. Mutants Y107A, Y240A and I262A lost or had very low responses to the tested drugs, the reduced responses could be due to altered expression, misfolding, impaired trafficking, or disrupted G protein coupling, and not necessarily to changes in ligand binding or activation alone. In particular, mutations located distal to the orthosteric binding pocket could be involved in allosteric communication, transmitting conformational changes from the ligand-binding site to intracellular regions that interact with G proteins. Some residues may act as allosteric microswitches, contributing to receptor activation by stabilizing active conformations or facilitating G protein engagement. Our TIRF results showed that mutants Y107A, Y240A and I262A exhibited surface expression levels comparable to the wild-type receptor (S1 Fig). However, further structural and biophysical studies may be required to delineate the specific roles of these residues in receptor activation pathways.

**Fig 6:**
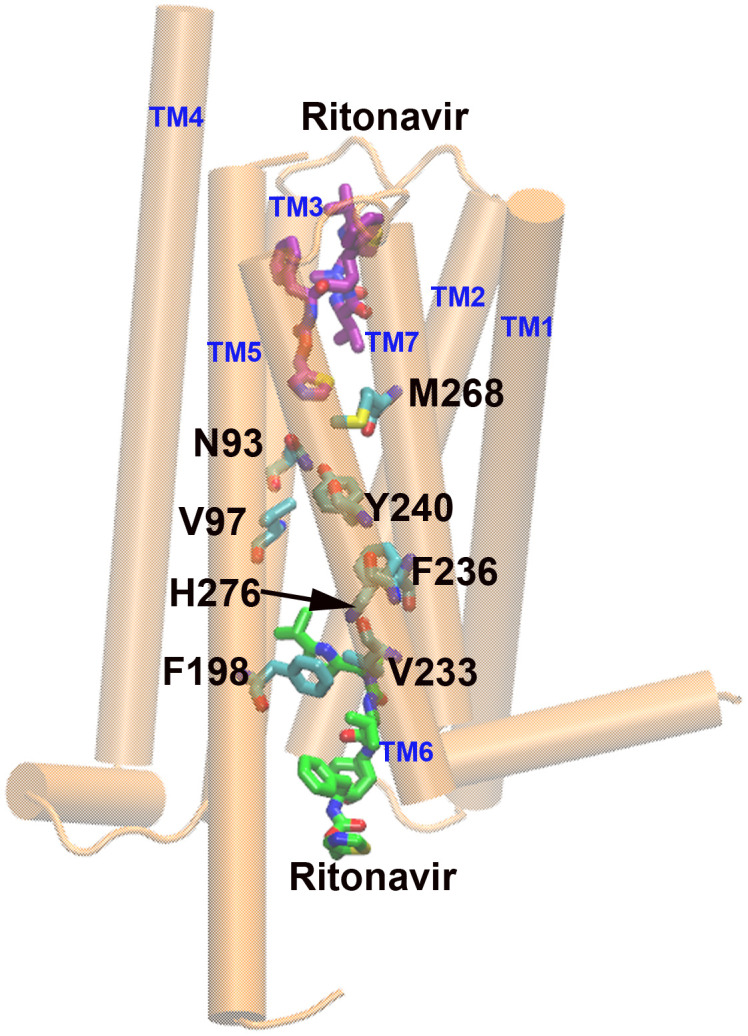
The upper and lower binding sites of ritonavir in hTAS2R14. hTAS2R14 is shown as cartoon colored in orange, ritonavir and critical interacting residues are shown as licorice colored by atom types.

Taken together, our results suggest ritonavir has two potential binding sites, orthosteric and allosteric sites in TAS2R14 (Fig 6). Although ritonavir is a relatively larger molecule than AA, FA, and compound 28.1, our docked model shows that it fits well into the allosteric site, which was further validated by our mutagenesis and functional results. Cryo-EM structures reveal that cholesterol binds to the orthosteric site of TAS2R14 [15,17]. While agonists (FA) can bind to both the orthosteric and allosteric sites [16]. Our studies suggest ritonavir may activate TAS2R14 through the orthosteric site, allosteric site, or both. The potential role of cholesterol in TAS2R14 activation by ritonavir needs to be further investigated. Future studies investigating whether the orthosteric and allosteric binding pockets contribute additively or synergistically to signal transduction could yield deeper mechanistic insights into bitter taste receptor activation.

It is worth noting that ritonavir has an unusual property of causing a persistent bitter taste in clinical settings. Unlike transient bitter compounds, the bitterness of ritonavir continues throughout treatment with Paxlovid (ritonavir combined with nirmatrelvir) and typically subsides only after discontinuation [24]. One possible explanation could be that ritonavir is taken up by taste receptor cells, where it remains bound to internal or allosteric sites of TAS2R14, leading to sustained activation of the bitter taste response. Further studies, including molecular dynamics simulations, are needed to elucidate the molecular mechanisms of receptor activation by this drug. This knowledge could be leveraged to design drugs that modulate taste perception or target the receptor’s roles in other physiological processes.

## Supporting information

S1 Fig Surface expression levels of T2R14 and selected mutants. (PDF)

S1 Raw data Excel file: T2R14 and mutants calcium mobilization data for Fig 3A and 4A. (XLSX)

## Abbreviations

TM: Transmembrane helix
LPV/r: Lopinavir/ritonavir
HIV: Human Immunodeficiency Virus
GPCR: G protein-coupled receptor
IFD: Induced fit docking
Cryo-EM: Cryo-electron microscopy
AA: Aristolochic acid
FA: Flufenamic acid
OA: Oleuropein aglycon
TIRF: Total internal reflection fluorescence
