# Supplementary material for: Molecular basis for the activation of the bitter taste receptor TAS2R14 by Ritonavir
Source: PLoS One. 2025 Sep 19;20(9):e0332704. doi: 10.1371/journal.pone.0332704 (PMC12448334; doi:10.1371/journal.pone.0332704)
Supplement: S1 Fig — (PDF) [file pone.0332704.s001.pdf]

Supplementary materials

Molecular basis for the activation of the bitter taste receptor TAS2R14 by Ritonavir

Jiao Wen[1], Xinyi Ma[1], Xinyi Zhou[1], R Charles Kissell[1], Yongcheng Lu[1], Yukyoung Kim[1], Young Seo Lee[1], Alice Lee[1], Shurui Chen[1], Keman Xu[1], Leigh D Plant[1], Meng Cui[1*]

[1]Department of Pharmaceutical Sciences and the Center for Drug Discovery, School of Pharmacy and Pharmaceutical Sciences, Bouvé College of Health Sciences, Northeastern University, Boston, MA 02115

* To whom correspondence should be addressed: m.cui@northeastern.edu (MC)

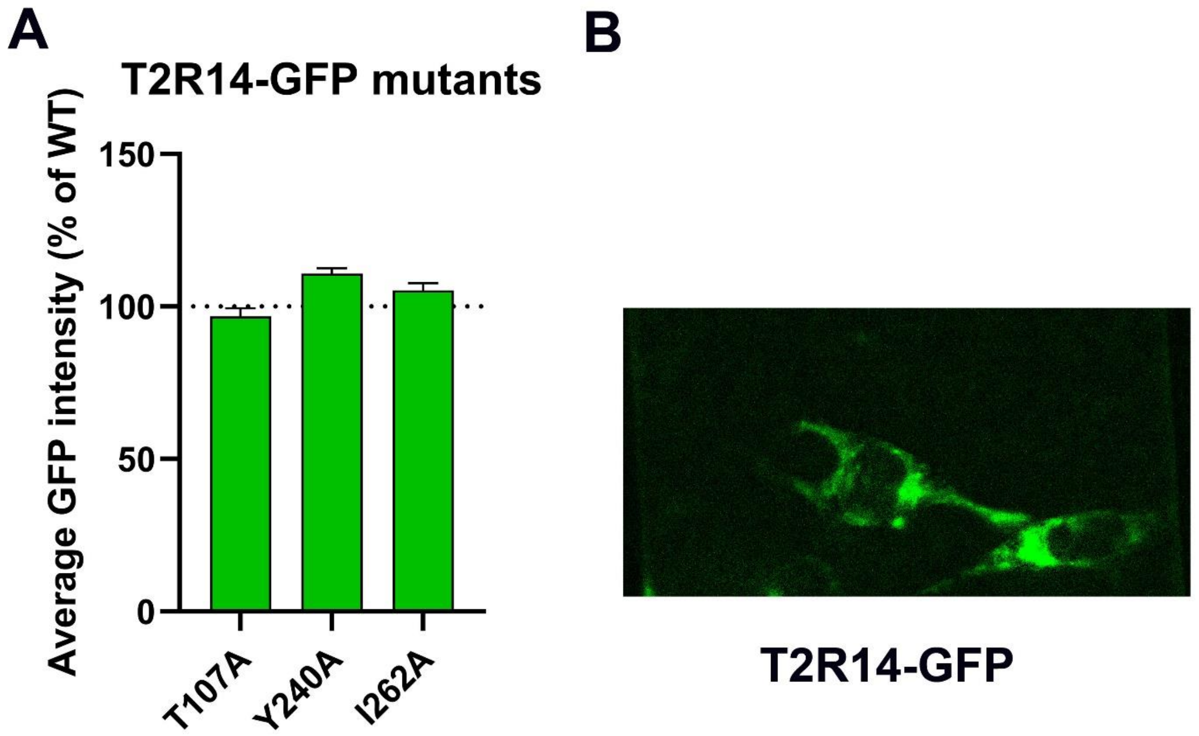

Figure S1. Surface expression levels of T2R14 and selected mutants. A. Relative surface expression levels of mutants T107A, Y240A and I262A compared to the T2R14 wild-type receptor. **B.** Representative TIRFM photomicrographs of HEK293T cells transfected with T2R14-GFP.
